# Nitrogen addition and harvest frequency rather than initial plant species composition determine vertical structure and light interception in grasslands

**DOI:** 10.1093/aobpla/plv089

**Published:** 2015-07-21

**Authors:** Ute Petersen, Johannes Isselstein

**Affiliations:** 1 Department of Crop Sciences, University of Göttingen, Von-Siebold-Str. 8, D-37075 Göttingen, Germany; 2 Present address: Johann Heinrich von Thünen Institute, Federal Research Institute for Rural Areas, Forestry and Fisheries, Institute of Climate-Smart Agriculture (AK), Bundesallee 50, D-38116 Braunschweig, Germany

**Keywords:** Canopy structure, dicots, light interception, monocots, permanent grassland, plant species composition

## Abstract

In biodiversity experiments based on seeded experimental communities, species richness and species composition exert a strong influence on canopy structure and can lead to an improved use of aboveground resources. In this study, we want to explore whether these findings are applicable to agriculturally managed permanent grassland. Vertical layered profiles of biomass, leaf area (LA) and light intensity were measured in a removal-type biodiversity experiment (GrassMan) to compare the canopy structure in grassland vegetation of different plant species composition (called sward types). Additionally, the altered sward types were subjected to four different management regimes by a combination of the factors fertilization (unfertilized, NPK fertilized) and cutting frequency (one late cut or three cuts). In spite of large compositional differences (ratio grasses : non-leguminous forbs : leguminous forbs ranging from 93 : 7 : 0 to 39 : 52 : 9), the vegetation of the same management regime hardly differed in its canopy structure, whereas the different management regimes led to distinct vertical profiles in the vegetation. However, the allocation of biomass in response to cutting and fertilization differed among the sward types. Vegetation dominated by grasses was denser and had more LA when fertilized compared with vegetation rich in dicots which merely grew taller. In functionally more diverse vegetation, light interception was not increased compared with vegetation consisting of more than 90 % of grasses in terms of biomass. Management had a much stronger influence on structure and light interception than plant species composition in this grassland experiment.

## Abstract (precis)

Recent biodiversity experiments using sown plant communities suggest a positive effect of plant species diversity on ecosystem functioning and resource use. However, are these experimental results applicable to agriculturally managed grassland? We analysed vegetation structure and light interception in managed grassland in which species composition had been manipulated by herbicides. We expected the functionally more diverse plots (grasses and forbs in equal amounts) to better intercept the light than plots containing more than 90% grasses due to an optimal arrangement of leaves in space. However, management (fertilization and mowing regime) had a much stronger influence on structure and light interception than plant species composition.

## Introduction

Canopy structure, the 3D arrangement of all aboveground plant components in space, is quite diverse in grasslands, despite their relatively low canopy height compared with woody vegetation (Körner 1994). There are not only species with horizontally arranged leaves and species with steep-angled leaves; there are also small and tall species leading to several distinct growth forms (Liira and Zobel 2000).

The determinants of canopy structure in permanent grasslands have been the subject of many scientific studies. Most of these studies have included some kind of classification according to the growth form of the different plants (e.g. Liira and Zobel 2000). The density, total plant height and vertical distribution of leaf area (LA) and biomass in a grassland canopy are influenced by water and nutrient content of the soil as well as by the frequency and type of biomass removal (pasture vs. meadow) (Werger *et al*. 1986; Mitchley and Willems 1995; Liira and Zobel 2000; Lienin and Kleyer 2012). Further, research on the leaf area index (LAI) of single species has been conducted (Sheehy and Cooper 1973; Sheehy and Peacock 1977; Haase *et al*. 1999; Cheng *et al*. 2009) with early studies by Brougham (1958) on LAI and light interception, emphasizing the importance of different leaf angles in a grassland canopy.

Within the still expanding research area on investigating the relationship between biodiversity and ecosystem processes, vertical structure and LAI in grasslands have generally been analysed in relation to species richness. On the one hand, the vertical structure has been shown to exert a strong influence on biodiversity due to its impact on light availability (Mitchley and Willems 1995; Lamb 2008), whilst on the other hand, species richness and species composition can also influence vertical structure. Indeed, Spehn *et al*. (2000), Fridley (2003), Lorentzen *et al*. (2008) and Vojtech *et al*. (2008) all found a positive effect of species richness on LAI and biomass, and concluded that resource partitioning of light and complementary use of aboveground space plays a role in canopy development, especially in environments where nutrients are not limiting. Since space *per se* is not a resource plants actively compete for (Wilson *et al*. 2007), the term ‘complementary use of space’ here in this article refers to the arrangement of leaves in space to indicate the effectiveness of light interception. However, the previously mentioned authors (Spehn *et al*. 2000; Fridley 2003; Lorentzen *et al*. 2008; Vojtech *et al*. 2008) used seeded, experimental plant communities and the applicability of results from these young, immature plant communities on real-world ecosystems is currently debated (Schmid 2002; Thompson *et al*. 2005; Hector *et al*. 2007; Mokany *et al*. 2008; Jiang *et al*. 2009). One criticism is that many of these types of experiments are not managed according to common agricultural practices in managed European grasslands (several cuts/grazing events per year and appropriate fertilization) (Wrage *et al*. 2011), thus lowering the transferability of results obtained from studies of experimental plant communities to actual managed grasslands. In addition, biodiversity experiments are generally designed with only a few species used (up to five, e.g. Vojtech *et al*. 2008; Nyfeler *et al*. 2009) or their species richness levels follow a logarithmic scale (1, 2, 4, 8, 16, 32 species, e.g. Spehn *et al*. 2000; Roscher *et al*. 2004; Tilman *et al*. 2012). Hence, only two of these experimental species richness levels (8 and 16 species) are comparable with species richness in managed grasslands in Europe where species richness ranges from 7 to 25 species per square metre (Pywell *et al*. 2007; Scimone *et al*. 2007). Therefore, the applicability of the experimental results to managed grasslands in Europe is questionable. Another approach, and a reasonable complement to biodiversity experiments, is removal experiments, where one part of the vegetation (most of the time one or several functional groups) is removed manually or by herbicide application (Díaz *et al*. 2003; McLaren and Turkington 2010; Petersen *et al*. 2012).

In 2008, we set-up a removal experiment and manipulated permanent grassland by herbicide application to obtain three different types of grassland vegetation (hereafter referred to as sward types); vegetation containing, in terms of biomass, <5 % dicots (–Dic); vegetation with a ratio monocots : dicots = 50 : 50 (–Mon) and untreated control vegetation (Co; for further details see Petersen *et al*. 2012 and the Methods section). With our experiment, we tried to close the gap between low (<10) and high (>30) species numbers per experimental unit. In the first year after the set-up, the grassland vegetation of the experimental plots had a species richness ranging from 7 to 16/m^2^ or 16–28 per experimental unit (here 225 m^2^). Roy (2001) outlined in his meta-analysis of biodiversity–ecosystem functioning studies that the largest effects were found at species numbers up to four. Clearly, we have more than four species in this experiment and therefore expect species richness effects on canopy structure and light interception to be small. However, we predict that over and above species richness functional and compositional differences amongst the sward types due to different growth forms are important factors to be taken into account. We hypothesize that the results of the seeded experiments mentioned above (Spehn *et al*. 2000; Fridley 2003; Lorentzen *et al*. 2008; Vojtech *et al*. 2008) are transferrable to managed grassland. Hence, the following two hypotheses should apply to the vegetation of our experimental plots: (a) depending on the fertilization and cutting regime, the species composition of the vegetation determines its vertical structure and LAI and (b) coexistence of different growth forms (i.e. different leaf angles) in a functionally more diverse grassland vegetation leads to better light interception compared with grass-dominated vegetation, because different growth forms lead to a more efficient arrangement of leaves for light interception.

## Methods

### Biodiversity experiment

The grassland management (GrassMan) experiment is a removal-type biodiversity experiment incorporating different management intensities. It was established in 2008 on permanent grassland between Silberborn and Neuhaus in the Solling Uplands, Germany (51°44′53″N, 9°32′42″E, 490 m above sea level (a.s.l.)). Prior to the set-up of the experiment, the grassland had been used as summer pasture for cattle. The area has a mean annual temperature of 6.9 °C and mean annual precipitation of 1028 mm (German Meteorological Service, DWD 1960–91, station Silberborn-Holzminden, 440 m a.s.l.). The soil type of the experimental area was classified as Inceptisol, more specifically as Haplic Cambisol (Keuter *et al*. 2013) and its vegetation was a nutrient-poor *Lolio-Cynosuretum* Br.-Bl. et De Leeuw 1936.

GrassMan is a three-factorial experiment (Table 1). The three sward types were obtained by a single herbicide application in July 2008 targeting dicotyledonous and monocotyledonous species, respectively. For the scheduled harvests and yield determination in 2009 we used a Haldrup^®^ forage combine harvester with a cutting height of 7 cm.

**Table 1.** Experimental factors and treatment levels of the GrassMan experiment. The acronyms of the different treatments are generated by combination of the factor level abbreviations in the order sward–utilization–nutrients, e.g. –Dic1x = dicot-reduced sward, cut once, no fertilization. *N fertilizer: calcium ammonium nitrate N27, P&K fertilizer: Thomaskali^®^ (8 % P_2_O_5_, 15 % K_2_O, 20 % CaO).

Factor	Level	Abbreviation
Sward type	1.1 untreated control sward	Co
	1.2 dicots reduced	–Dic
	1.3 monocots reduced	–Mon
Utilization	2.1 1 cut (July)	1
	2.2 3 cuts (May, July, September)	3
Nutrients	3.1 no fertilization	*X*
	3.2 180/30/100 kg NPK ha^−1^ year^−1^ *	NPK

The combination of all factors and levels resulted in 12 experimental treatments, which were replicated six times. The treatment names were generated by a combination of the abbreviation of experimental factor levels in the order sward–utilization–nutrients, e.g. Co3x = control sward, cut three times, no fertilization. The two factors simulating the intensity of agricultural management, i.e. fertilization and cutting, will later be referred to as management regimes. To account for potential spatial gradients, the 72 experimental plots were arranged to form a Latin Rectangle with the upper part bordering a forest sloping gently (<5 % slope) towards the lower rows. Two columns form one block. A detailed description of the experiment can be found in Petersen *et al*. (2012).

### Biomass and measurements for canopy structure

To describe the canopy structure, we used the vertical layer-wise distribution of biomass within the vegetation. Here, biomass comprised the total aboveground biomass of a defined area, not just the part which was harvested for agricultural purposes (yield). The spatial arrangement of biomass was analysed by stratified clipping (Monsi and Saeki 1953; Sheehy and Cooper 1973), i.e. harvesting the biomass in 10 cm thick layers along the canopy. Prior to each of the scheduled harvests in 2009 (May, July and September 2009), we pegged three randomly selected 15 × 15 cm quadrats per plot (=subsample). The generative growing points, i.e. the buds of all dicotyledonous species, were counted before cutting the subsample at the ground level. The cut bunch of grass was separated into 10 cm layers starting at the ground level, i.e. separated into volumes of 15 × 15 × 10 cm except for the lowest layer which was 7 cm thick, corresponding to the cutting height of the forage harvester to ensure comparability of total biomass and harvested yield at the different harvest dates. The material of each layer was placed in a plastic bag and frozen before further processing. The three subsamples of each plot corresponding to the same layer were combined to one sample and sorted to the three functional groups [grasses, non-leguminous forbs and leguminous forbs (later called legumes)] and dead plant material (=standing litter). The sorted material was dried at 105 °C for 48 h and weighed. Additionally, the number of grass tillers and vegetative growing points of clover in the lowest layer were counted. Both vegetative and generative growing points will hereafter be referred to as buds.

From these data, we calculated the harvest index, i.e. the ratio of economic yield to biological yield or total aboveground biomass. This index originates from crop yield calculations, e.g. grain mass versus total biomass of cereals (Donald 1962). Moreover, we determined the centre of gravity (*c*) per plot according to Spehn *et al*. (2000) as a measure of vertical biomass density.

$$c=\frac{1}{m_{t}}\times \overset{n}{\underset{l=1}{\sum }}(m_{l}\times h_{l})$$

where *m_l_* is the biomass of one layer, *h_l_* the corresponding average height of this layer (here 10 cm or 7 cm) and *m_t_* the total biomass of the profile. The centre of gravity indicates the canopy height (in cm), around which most of the biomass is concentrated. The centre of gravity is raised in very tall canopies compared with short canopies or in canopies of equal height where the biomass is distributed more evenly across the different canopy layers, i.e. where the vertical profile has a convex rather than a concave shape.

### Leaf area index and light measurements

The LAI was determined at the time of peak standing biomass at the second harvest (first harvest in the 1-cut treatments) in July. The LAI of the subsamples was not measured directly but calculated by multiplying the specific leaf area (SLA) of each biomass fraction (stems and leaves of grasses, non-leguminous forbs and legumes) by the corresponding biomasses harvested by the stratified clipping. For the determination of SLA, a mixed sample of fresh plant material from each block separated according to cutting regime was taken, put into a cool box and transferred to the laboratory immediately. Legumes were collected separately from plots belonging to the same utilization × nutrient combination, since preceding tests had shown that in contrast to non-leguminous forbs and grasses, the SLA of the legumes (consisting of more than 90 % *Trifolium repens* L.) was influenced not only by cutting regime but also by nutrient supply. We followed the protocol for determining SLA by Garnier *et al.* (2001) and kept the material damp and cool before scanning 20–30 subsamples of stems and leaves of each functional group using the software WinRhizo (Version 2007 d, Regent Instruments, Inc., Quebec, Canada). The scanned samples were dried at 105 °C for 48 h before weighing. From a regression between dry biomass and scanned leaf area, SLA was calculated. Owing to the large heterogeneity of stem diameters in legumes and non-leguminous forbs, the goodness of fit of the mass–area regression was more variable for all forbs than for grasses (adjusted *R*^2^ between 0.68 and 0.95 for forbs, from 0.85 to 0.99 for grasses). Additionally, the stem data of non-leguminous forbs from one block were missing completely and would have had to be estimated from the other data. In all plots containing a certain amount of forbs, this would have led to a larger variability in estimated LA than in plots with more than 90 % grasses. To eliminate these biased values, we decided to exclude all LA calculated from stem biomass from further analyses and only used LA from leaf blades. Thus, the leaf area ratio (LAR, leaf area per sample dry weight, mm^2^ mg^−1^) was not based on the total leaf area (Ryser and Lambers 1995) but on the leaf area of laminae, likewise the LAI (called LAI leaves from here on).

Prior to each harvest, the light interception was measured using the SunScan canopy analysis system (Typ SS1; Delta-T Devices Ltd, Cambridge, UK) with a portable reference sensor directly above the canopy of each plot. The photosynthetically active photon flux density (PPFD) was measured in vertical proﬁles from different heights within and above the canopy (0, 7, 17, 27 cm and so on up to 117 cm in the tallest canopies) each with three replicates per plot, corresponding to the biomass layers in the subsamples cut afterwards.

The light extinction coefficient *k* was calculated following the Lambert–Beer extinction law *I* = *I*_0_ × e^−*k*LAI^ by using the determined LAI of the leaves and light intensity at the ground level (Saeki 1959; Spehn *et al*. 2000), where *I* = PPFD at the ground level and *I*_0_ = PPFD above the canopy measured by the reference sensor. The extinction coefficient *k* does not only describe how readily the incoming light is intercepted (= absorbed or reflected back); it also gives a hint to the distribution of leaf angles within the canopy (Geyger 1977). The more vertically orientated the leaf blades are, the lower is their extinction coefficient and vice versa. The species richness and the functional diversity, i.e. the proportions of grasses, non-leguminous forbs and legumes, of the vegetation had been determined by vegetation relevés (9 m^2^) in May and August 2009. Since the three sward types Co, –Dic and –Mon differed significantly in their proportions of functional groups with grass contents of 70, 92 and 45 %, respectively, and with almost no legumes present in the –Dic-swards (Petersen *et al*. 2012), the sward types were taken as surrogates for functional diversity in this study with functional diversity increasing from –Dic via Co-swards to –Mon-swards.

### Statistical analysis

Univariate statistics were calculated in R version 2.12.2 (R Development Core Team 2011). Since in an analysis of variance (ANOVA), the order of explaining variables is important (see example given below), row and block were included first of all factors into all (general) linear models (GLM/LM) we applied on our data. With this order of factors, we were able to extract all environmental variability before assessing treatment effects. The effects of the experimental factors sward, utilization and nutrients on biomass, centre of gravity, harvest index, light interception and extinction, LAI and LAR were tested using a three-way ANOVA including all treatment factors and their interactions. For example harvest index = row + block + sward × nutrients × utilization (data from July harvest) and harvest index = row + block + sward × nutrients (May and September data). For biomass, LAI and LAR we further fitted alternative models including species richness instead of sward type, to compare the effects of species richness (one aspect of species composition) on our subplots with those obtained on seeded experimental plots. The models were simplified by dropping insignificant interactions and factors (apart from row and block) following the instructions given in Zuur *et al*. (2009) using the Aikaike Information criterion (AIC) to detect the best fitting models. For comparison of means of several factor levels, we used Tukey's ‘Honest Significant Difference’ method (Miller 1981) with a confidence level of 0.95. To ensure better comparability of the layer-wise distribution of LA and biomass, these data were expressed as the proportion of the total LA or biomass. We applied general linear models with the binomial error distribution on these proportional data. We compared the proportions of biomass in a given canopy layer in each of the sward types belonging to the same cutting and fertilization level. All layers were compared up to the ones where 80 % of total biomass or LA was reached. This was the layer 37–47 cm for biomass and 27–37 cm for LA. The models included the experimental factors or, to compare the means of single treatments, the 12 treatments themselves. Overdispersion larger than 1.5 (i.e. the variance in a GLM with Poisson distributed errors was at least 1.5 times larger than the mean) was corrected by multiplication of standard errors with the overdispersion parameter Φ, using a quasi-GLM model or a model with negative binomial distribution of errors (Zuur *et al*. 2009). Normal distribution and homogeneity of the residuals were inspected using QQ plots and conditional boxplots of the residuals against all factor levels as suggested by Anscombe (1973) and Zuur *et al*. (2010). Heterogeneity of variances was corrected using general least square (GLS) models including the ‘varIdent’ variance structure (different variance per factor level, this R-function is found in the ‘nlme’ package (Pinheiro *et al*. 2009). Necessary data transformations are indicated in the results.

## Results

### Canopy composition and structure

The botanical and functional composition of the three sward types showed a distinct variation in the buds and tillers per square metre (Table 2). Since there were almost no legume buds in the –Dic-swards, the variance of the depending variable ‘legume bud number’ was close to zero in this sward type. Consequently, this sward type had to be excluded from all legume analyses. The three-way ANOVAs and linear contrasts (data not shown) underlined the effects of the herbicide treatment, i.e. the sward types, on bud and tiller numbers. At all harvest dates, the bud numbers of legumes and non-leguminous forbs averaged across all cutting and fertilization levels differed significantly among the sward types (*t*_(20, α)_ ≥ 2.853, *P* ≤ 0.0098, linear contrasts in LM and GLS), except for the May harvest, when legume bud numbers were almost equal in the Co- and –Mon-swards. The impact of fertilization on bud numbers increased over the course of the year. No effect was found in May and July, while in September the bud numbers of legumes had been reduced by fertilization (*z*_(11, α)_ = 2.568, *P* = 0.010, linear contrasts in GLM with negative binomial error distribution) and non-leguminous forb bud numbers were significantly lower in fertilized plots (*t*_(20, α)_ = 2.789, *P* = 0.011, linear contrasts in LM). The number of grass tillers in May, however, was increased by fertilization (*t*_(22, α)_ = 2.401, *P* = 0.025, LM, response variable raised to the power of 0.25). Significant sward effects were only found in May and September (ANOVA, *F*_May(2, 22)_ = 21.62, *F*_Sept(2, 19)_ = 14.11, *P* < 0.001).

**Table 2.** Vegetative and generative buds/number of tillers per m^2^ in the experimental treatments at each harvest 2009. Mean and standard deviation for grasses, median and median deviation for non-leguminous forbs and legumes, *n* = 6. For treatment abbreviation, refer to Table 1.

	Grass	Non-leguminous forbs	Legumes
May 2009			
Co3x	10 173 ± 3071	504 ± 222	238 ± 148
Co3NPK	11 291 ± 2922	593 ± 74	326 ± 230
−Dic3x	13 111 ± 5150	170 ± 111	0 ± 0
−Dic3NPK	18 304 ± 4632	200 ± 148	0 ± 0
−Mon3x	7948 ± 2532	1171 ± 164	200 ± 111
−Mon3NPK	8605 ± 3044	897 ± 89	252 ± 111
July 2009			
Co1x	5563 ± 3504	511 ± 126	82 ± 67
Co1NPK	4203 ± 2733	474 ± 200	45 ± 45
Co3x	8763 ± 3636	771 ± 133	126 ± 52
Co3NPK	7607 ± 5882	407 ± 89	111 ± 111
−Dic1x	5600 ± 2878	134 ± 112	0 ± 0
−Dic1NPK	6360 ± 4627	148 ± 104	0 ± 0
−Dic3x	6425 ± 996	289 ± 89	0 ± 0
−Dic3NPK	10 286 ± 5258	134 ± 111	0 ± 0
−Mon1x	5869 ± 2463	875 ± 327	348 ± 185
−Mon1NPK	2768 ± 1075	1252 ± 415	119 ± 89
−Mon3x	4615 ± 1569	971 ± 319	274 ± 59
−Mon3NPK	11 477 ± 3810	704 ± 156	82 ± 52
Sept 2009			
Co3x	9353 ± 1191	778 ± 163	141 ± 104
Co3NPK	10 062 ± 5411	289 ± 119	156 ± 82
−Dic3x	13 953 ± 3973	223 ± 44	0 ± 0
−Dic3NPK	11 916 ± 6993	96 ± 96	0 ± 0
−Mon3x	8479 ± 2762	1304 ± 282	400 ± 111
−Mon3NPK	8721 ± 4448	608 ± 363	341 ± 282

**Figure 1.**
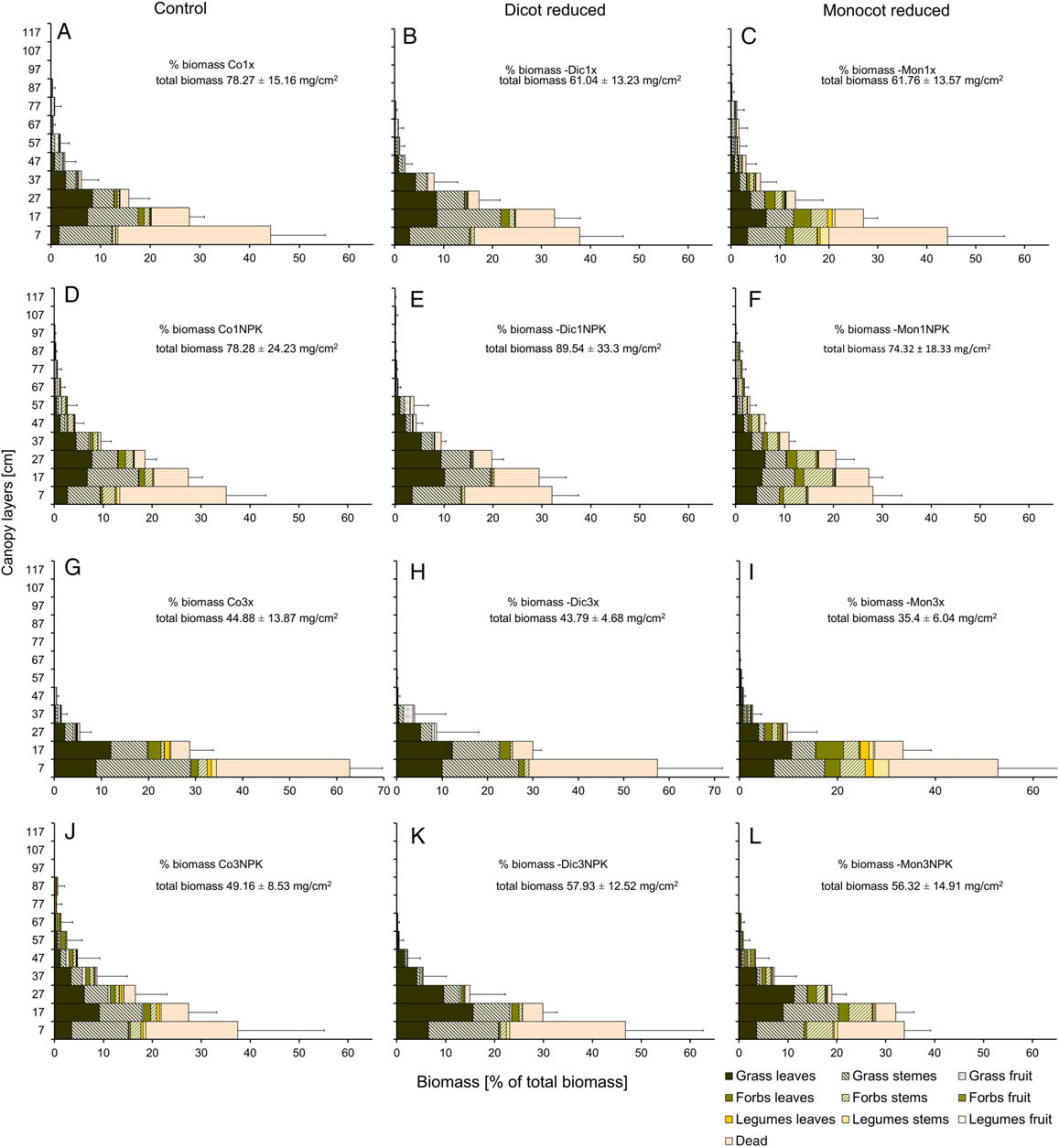
Vertical distribution of the different functional fractions of biomass for all 12 experimental treatments (July 2009). Given are means of total biomass, biomass of each fraction per layer and standard deviations of each layer, *n* = 6. For abbreviations of treatments, refer to Table 1.

Total biomass of the July canopy profiles belonging to the same management regime did not differ among the sward types (means and standard deviations displayed in Fig. 1). Likewise, species richness had no effect on biomass when included as first experimental factor in a GLS with different variances per fertilization level. However, the two manipulated sward types gained significantly more biomass from fertilization than the Co-swards (interaction sward × nutrients, *t*_(51, α)_ = 2.051, *P* = 0.045 for –Dic and *t*_(51, α)_ = 2.345, *P* = 0.023 for –Mon-swards, respectively, GLS allowing for different variance in each cut × nutrient combination). Averaged across all management treatments, the –Mon-swards had produced the least biomass (*t*_(51, α)_ = 2.49, *P* = 0.015).

All three experimental factors influenced the vertical structure of the vegetation in the subplots, as demonstrated by the layered diagrams in Fig. 1. The outer shape of the layered diagrams (a line from top to bottom connecting the outer edges of the horizontal bars) was defined by the management. The biomass diagrams of unfertilized plots had a concave shape, the fertilized ones a more convex shape, especially close to the ground. The tallest canopy height was determined by the cutting regime. Significant sward effects were only found within the same management regime. Most of the structural differences, i.e. significant differences in biomass proportions per 10 cm layer due to the factor ‘sward type’, were observed between –Mon (Fig. 1C, F, I and L) and –Dic-swards (Fig. 1B, E, H and K) and they were confined to two of the medium layers (7–17 and 27–37 cm). Differences were either found in fertilized plots belonging to different sward types or when comparing the change in vertical structure between different management regimes (fertilized vs. unfertilized, cut once vs. cut thrice). Under fertilization, both Co- and –Mon-swards had increased their biomass proportions in layer 27–37 cm significantly more than the –Dic-swards, by 7.2 % (Co) and 4.9 % (–Mon) compared with 1.4 % in the –Dic-swards (sward × nutrient interaction in Co-swards cut thrice, *t*_(16, α)_ = 2.208, *P* = 0.004 and –Mon-swards cut once *t*_(16, α)_ = 2.758, *P* = 0.006). Compared with the plots cut once, the proportion of biomass belonging to layer 7–17 cm in the three-cut regime increased significantly more in the –Mon-swards (5.3 %) than in the –Dic-swards (1.1 %) (significant sward × cut interaction, *t*_(45, α)_ = 2.321, *P* = 0.020). The –Mon-swards cut once had allocated significantly less biomass, on average 24 %, to layer 7–17 cm than the –Dic-swards (*t*_(18, α)_ = −2.118, *P* = 0.034, Fig. 1B, C, E and F), where around 30 % of total biomass belonged to this layer. Above all, fertilization led to significant relocation of biomass from the lowest into higher (>17 cm) canopy layers in Co- and –Mon-swards, whereas the –Dic-swards hardly changed their vertical biomass distribution under nutrient enrichment.

All diagrams (Fig. 1A–L) show that a large proportion of the total biomass was concentrated in the lowest layer and remained as stubble after the harvest, which led to harvest indices from 36 ± 5.6 up to 73.5 ± 6.7. Still, the harvest indices in July were clearly larger than in May (between 17.9 ± 2.6 and 27.8 ± 1.5) and September (ranging from 33.7 ± 11.2 to 40 ± 13.8). The sward type was not responsible for any variation in harvest indices, they were mainly influenced by fertilization (ANOVA with arcsine-square root transformation, *F*_(1, 24)_ = 7.31, *P* = 0.012 in May, *F*_(1, 50)_ = 35.76, *P* < 0.001 in July) and, in July, by the cutting frequency (*F*_(1, 50)_ = 25.10, *P* < 0.001). The third regrowth in September had similar harvest indices across all experimental treatments.

As already indicated in the shapes of the layered diagrams, the location of the centre of gravity was raised by fertilization (linear contrasts in LM with log transformation, *t*_(52, α)_ = 6.180, *P* < 0.001) and lowered by a higher cutting frequency (*t*_(52, α)_ = 6.827, *P* < 0.001). The centre of gravity was located between 7.5 ± 1.0 cm (Co3x) and 19.2 ± 1.3 cm (–Mon1NPK) above the ground. In the –Mon-swards the centre of gravity was located ∼1 cm further up in the canopy than in the other two sward types, although this effect was not significant.

**Figure 2.**
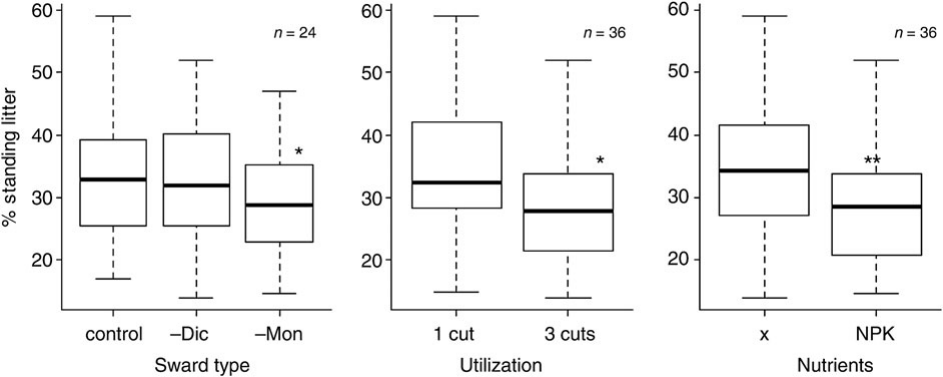
Proportion of standing litter based on total biomass found in the canopy profiles shortly before the harvest in July 2009 depending on experimental factors. The boxes show the median and first and third quartiles. For abbreviations of treatments, refer to Table 1. Asterisks denote significant differences of factor levels compared with the reference level (sward control, utilization 1/year, fertilization x) in a GLM with negative binomial error distribution (link: logit). **P < 0.01, *P < 0.05.

The amount of standing litter within the vegetation (Fig. 2) mainly depended on age and nutrient status of the plots. The high cutting frequency with an early first cut (plots belonging to the three-cut regime) and fertilization significantly decreased the ratio of dead to green plant material. However, the amount of grasses present in the vegetation also tended to influence the amount of litter (analysis of deviance of a linear model with negative binomial error distribution *P* = 0.07). The –Mon-swards, containing the least grasses also had the lowest amounts of standing litter.

### Leaf area index and light distribution

The total LAI of the vegetation was neither influenced by the sward type nor the species richness within the same management regime. Just the ability to transform applied fertilizer into leaf area was significantly higher in the –Dic-swards than in the Co-swards (interaction sward × nutrients, *t*_(49, α)_ = 2.517, *P* = 0.015, GLS allowing for different variances per fertilization level). The LA profiles had a concave–convex shape across all treatments due to a concentration of LA in canopy layers from 7 to 27 cm. The only significant sward influences on the vertical LA distribution were found in fertilized experimental treatments. In the fertilized plots cut three times, we found a significantly higher proportion of LA in higher canopy layers in the –Mon than in the –Dic-swards (*t*_(43, α)_ = 2.047, *P* = 0.050). The lowest layer close to the ground had the lowest proportion of LA in the fertilized Co-swards of cut once, significantly lower compared with the –Mon-swards (*t*_(4, α)_ = 2.812, *P* = 0.048).

**Figure 3.**
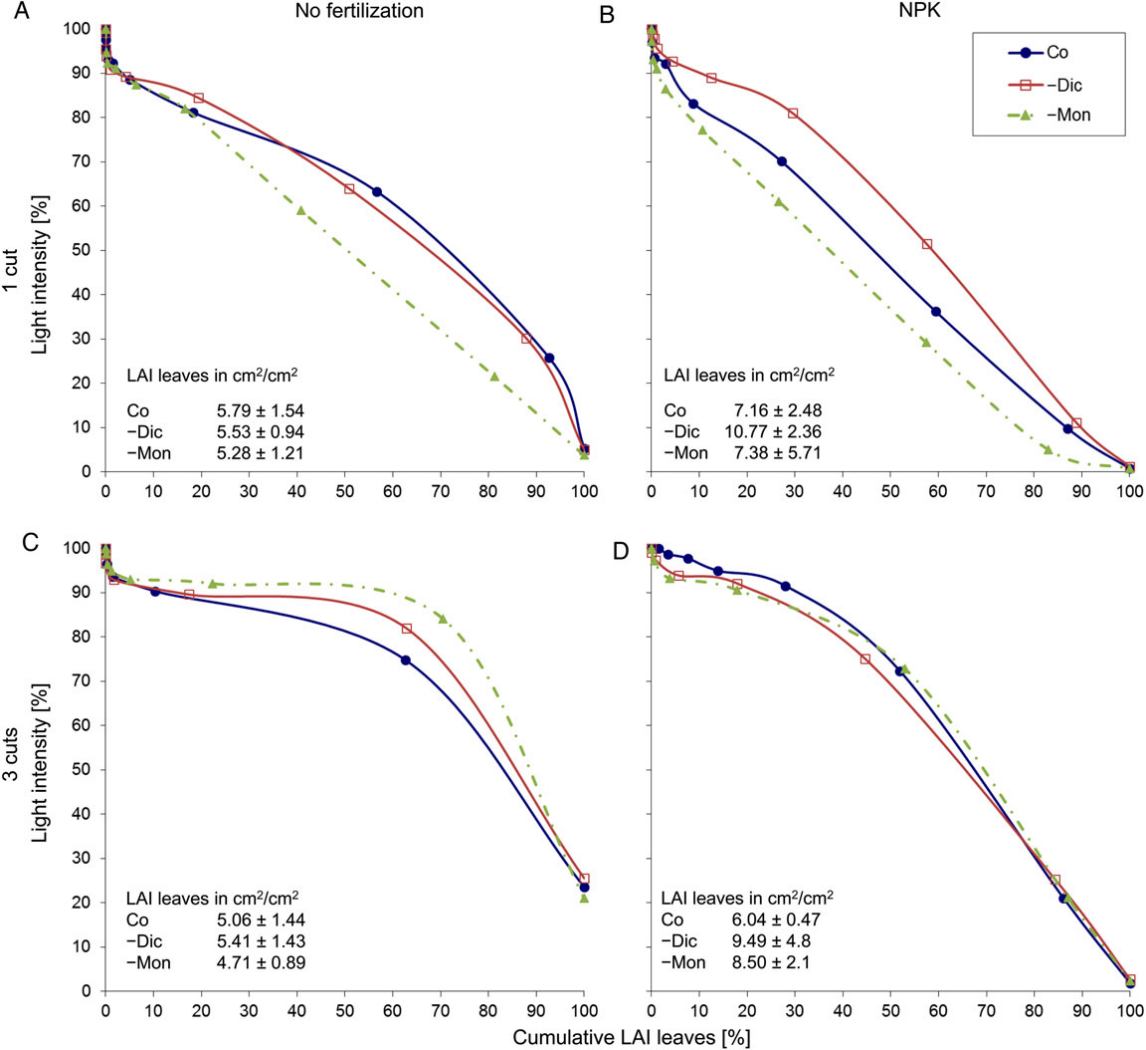
Means of LAI (leaves) and light intensity in relation to cumulative LAI (leaves) in the experimental treatments in July 2009 grouped by management regime (*A* = 1*x*, *B* = 1NPK, *C* = 3*x*, *D* = 3NPK). The sloping curves show the light intensity from above the canopy (0 % LAI) in 10 cm steps down to the ground (100 % LAI), *n* = 6.

A combination of values of light extinction and LA showed more pronounced differences between the three sward types (Fig. 3). The extinction curves all followed a distinct shape depending on cutting frequency. In the mature vegetation cut once, it took much less LA to intercept a certain amount of light compared with the vegetation of the plots in the three-cut regime. The –Mon-swards showed the strongest structural changes under management variation. In the plots cut three times without fertilization (Fig. 3C), the vegetation of the –Mon-swards tended to intercept the least light with 50 % of its LAI compared with the vegetation of the other sward types. In contrast, in the fertilized plots cut once (Fig. 3B), the vegetation of the –Mon-swards intercepted the most light (significantly more than the other two sward types, *P* ≤ 0.03, linear contrasts in LM).

**Figure 4.**
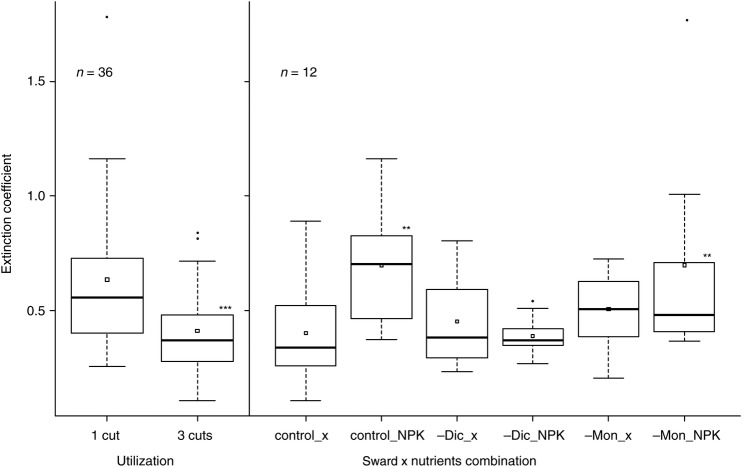
Extinction coefficient in the different experimental treatments in July 2009. Asterisks denote significant differences of factor levels compared with the reference level (sward control, utilization 1/year, control_ x). Interaction sward × nutrients (*F*_(2, 49)_ = 3.30, *P* = 0.045).

The extinction coefficient was lowered by a high cutting frequency (Fig. 4). In both Co- and –Mon-swards, it increased due to fertilization, whereas the –Dic-swards showed a similar light extinction in both nutrient regimes (sward × nutrient interaction in ANOVA, *F*_(2, 49)_ = 3.30, *P* = 0.045). In just a few of the fertilized Co-swards, the leaves grew mainly in wide angles (>45° between stem and leaf), indicated by an extinction coefficient *k* larger than 1. The vegetation in all other treatments showed an equal distribution of leaf angles apart from the vegetation in the –Dic-swards, which had mainly vertically orientated leaves (leaf angle <45°, 0.3 < *k* < 0.7). In spite of these different extinction coefficients, the light reaching the ground was similar in all sward types; it was only influenced by management regime (Table 3).

**Table 3.** Median and median deviations of light transmission (*I*/*I*_0_) per treatment at ground level in July 2009 with corresponding ANOVA table (adjusted for the factors row and block, data log transformed, *n* = 6).

	No fertilization (*x*)		Fertilized (NPK)	
	1 cut	3 cuts	1 cut	3 cuts
Control	0.05 ± 0.03	0.24 ± 0.04	0.01 ± 0.003	0.02 ± 0.01
−Dic	0.05 ± 0.03	0.26 ± 0.09	0.01 ± 0.002	0.03 ± 0.02
−Mon	0.04 ± 0.01	0.21 ± 0.08	0.01 ± 0.003	0.02 ± 0.02
	Df	MSq	*F*-value	*P*
Sward type	2	0.847	1.72	0.187
Utilization	1	30.296	61.43	<0.001
Nutrients	1	59.570	120.78	<0.001
Residuals	56	0.535

**Figure 5.**
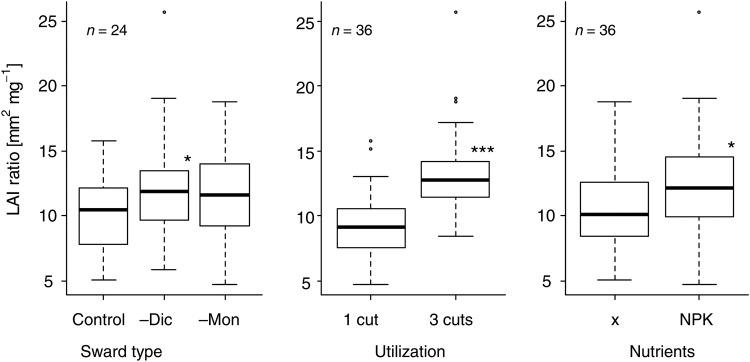
Leaf area ratio (median, first and third quartiles) per experimental factor in July 2009. For abbreviations of factors, refer to Table 1. Asterisks denote significant differences of factor levels compared with the reference level (sward control, utilization 1 cut, nutrients x) in a linear model (with log transformation of the response variable for comparison of treatments). ***P < 0.001, **P < 0.01, *P < 0.05.

The LAR was influenced mainly by management, but also by the amount of grasses present in a sward (Fig. 5). Overall, the –Dic-swards had a higher LAR than the Co-swards. The comparison of the single treatments, however, did not show any sward or species richness effects. The highest LAR was found in the fertilized –Dic-swards cut three times.

## Discussion

In this study we examined the canopy structure of permanent grassland under different management regimes with contrasting plant species composition of species from the same plant community. In accordance with many other publications, we consider the biomass or LA profiles as main indicators for vertical structure (e.g. Fliervoet and Werger 1984, 1985; Werger *et al*. 1986; Mitchley and Willems 1995). To complement the profiles, the following canopy variables or indices were used: centre of gravity, extinction coefficient representing the prevalent leaf angles, light extinction (as in Spehn *et al*. 2000), LAR and the total LAI (as in Fliervoet 1987). We aimed to compare our results with those obtained in seeded grassland vegetation commonly used in biodiversity experiments.

The species composition, described by the numbers of growing points of each functional group, was not stable over the whole season. Pavlů *et al*. (2006) also encountered fluctuating numbers of grass tillers, legume buds and non-leguminous forb plants when comparing vegetation density in mown and continuously grazed pastures. In our experiment, nutrient addition, especially in combination with defoliation, stimulated the formation of grass tillers while reducing the numbers of buds in non-leguminous forbs and legumes. Management is known for strongly influencing species composition and density in permanent grassland (Prins and Van Burg 1979; Oomes and Mooi 1981; Klimek *et al*. 2007), leading to a new equilibrium in plant species composition. Since we were especially interested in structural differences brought about by species composition, we had restricted our analyses to one growing season.

In spite of the differences in species composition and the different growth forms, the sward types hardly showed any structural differences within one management regime. As also reported by Werger *et al*. (1986), who analysed different permanent grassland types in the Netherlands, the canopy form (from concave via linear up to convex) was largely determined by the nutrient status and the productivity of a plot. Nutrient addition generally elongated the canopy and raised the centre of gravity. However, the reaction to fertilization differed significantly among the sward types. The canopies of –Mon-swards grew taller after fertilization than –Dic-swards which remained largely unchanged in their vertical structure. This shift of biomass into upper canopy layers can be explained by the increased presence of tall species with leafy stems. This trait combination is typical for competitive species, which increase their biomass after nutrient addition and low levels of disturbance in natural grassland communities (Liira and Zobel 2000; Gross *et al*. 2007). In the –Mon-swards the total amount of these tall species was considerably higher than in the –Dic-swards (see Petersen *et al*. 2012) since they hosted apart from tall grasses also tall dicots with leafy stems (e.g. *Rumex acetosa* L., *Achillea millefolium* L.). Consequently, the biomass shift was much more pronounced compared with the –Dic-swards. Spehn *et al*. (2000) likewise observed a raised centre of gravity in functionally more diverse vegetation in seeded experimental plots.

The harvest index was not improved by functional diversity; it was only influenced by management. Especially the young (cut in May) or regularly cut short vegetation (in September, the vegetation only had a canopy height of around 20–25 cm) had not developed a distinct structure, which consequently could not have influenced the harvest index; a result which was also found by Liira and Zobel (2000). Only tall, mature grassland could develop a clear vertical structure they reported. All young or recently cut grassland vegetation is too short to develop a canopy with distinct layers, i.e. in all layers there is roughly the same amount of biomass. This was in line with our observations. The tillering of grasses stimulated by the first cut in May led to a dense canopy layer close to the ground which lowered the harvest index in the regrowth in July especially in –Dic-swards compared with the other sward types.

The amount of standing litter in the vegetation strongly increased due to a late cut without fertilization (1x regime) is also encountered by Marriott *et al*. (2005) on grassland vegetation in Scotland. However, the functional composition of the vegetation also influenced the amount of standing litter. The more grasses that were present; the more dead biomass was found. This correlation was also found by Fliervoet and Werger (1985) for Calthion grasslands in the Netherlands. Since the –Dic-swards were quite dense and consisted almost completely of grasses (=leafy stem species), they had the most standing litter. The fact that by the beginning of July most of the –Dic-swards were not standing upright any more increased the light deficiency and stimulated the death of leaves close to the ground.

The reason for the significant LA changes in grass-rich vegetation after fertilization lies in the different growth physiology of grasses compared with dicots. If they are fertilized, grasses increase their leaf area a lot more than dicots (Körner 1994). The LAI ratio underpinned these findings. The more grasses the vegetation contained, the higher was its LAR.

In this study, hypothesis (a) was only partly supported. We did find a strong influence of management on the vertical structure, but species composition effects were negligible. The location of light interception and hence the shape of the light intensity curves were mainly defined by the cutting regime. The different light extinction coefficients of the sward types were obviously due to differences in leaf angles. Light extinction is influenced by the vegetation's density but mainly by the angles of leaves with grassy vegetation having a lower extinction coefficient (0.3 < *k* < 0.7) than vegetation containing a higher percentage of dicots (*k* > 1.0, Geyger 1977). Fertilization and a late cut increased leaf size and biomass density, visible by a stronger light interception in upper canopy layers in –Mon-swards. Since fertilization did not change the leaves’ angles, the –Dic-swards had a lower extinction coefficient in spite of a larger LA, corroborated by Brougham's (1958) findings. Even though most of the flat-lying plants (= horizontal leaves from ∼17 cm downwards) were found in –Dic-swards, this angle effect was still visible. Fliervoet and Werger (1984) also mentioned the inclination and vertical distribution of leaves as the main factor determining the light interception in the permanent grassland they analysed.

The absolute light interception was similar in all plots of the same management regime. The hypothesized positive effect of species or functional diversity on light interception, as Spehn *et al*. (2000) found in seeded grassland vegetation, could not be confirmed. One reason for this discrepancy could be the different age of the vegetation. The grassland vegetation had been sown 2 years previous to the experiment and especially the vegetation consisting only of grass species had not even reached full ground cover. The vegetation in the GrassMan experiment was much older and had recovered completely from the herbicide treatment in the previous year in terms of cover and biomass (Petersen *et al*. 2013). Even though we had a contrasting species composition in terms of monocot to dicot ratio, these differences did not lead to structural or functional differences. The vegetation in plots cut three times may have been disturbed too often to build up a distinct structure as described for managed permanent grassland by Liira and Zobel (2000).

The fact that the light interception efficiency of our grassland ecosystem was not influenced by compositional differences [hypothesis (b)] is possibly due to its relatively high species number compared with experimental, seeded grassland. As Schwartz *et al*. (2000) and Waide *et al*. (1999) pointed out, most of the ecosystem processes are saturating at low species numbers; Roy (2001) had summarized in his meta-analysis that, in many studies, five plant species were enough to reach 90 % of the net primary productivity of the examined vegetation type. Since the vegetation in our experiment consisted of at least seven species per square metre, it was not at all surprising that the light interception, the ecosystem function analysed here, was not limited by the species number. A mixture of just two productive grass species achieves a denser filling of the aboveground space and hence a higher light interception compared with a monoculture as shown by Vojtech *et al*. (2008). Consequently, the efficient light interception in the –Dic-swards consisting of at least five grass species and small amounts of two or more dicot species is not surprising. In addition, the broad range of structural and functional diversity among monocots and dicots in this study may have been more important for other ecosystem functions (e.g. nutrient acquisition) than for light interception. Griffin *et al*. (2009) suggest that functional diversity can even have adverse effects on different ecosystem functions.

## Conclusions

Above all, management had a much stronger influence on structure and light interception than species composition in this grassland experiment. Compositional and functional differences turned out to be important only if different management intensities in terms of fertilization and cutting frequency were compared. Then different physiological and morphological traits of dicots and monocots became visible, leading to a slightly different structural development of the vegetation, whereas no effects of species richness were found. Hence, the strong effects due to species composition found in experimental, seeded grassland vegetation cannot be transferred directly to managed permanent grassland. Obviously, even in plots with a low number of species (7/m^2^) the structural and functional diversity was high enough to ensure an optimal arrangement of leaves in space to fully capture the incoming light.

## Sources of Funding

This study, funded by the Ministry of Science and Culture of Lower Saxony and the ‘Niedersächsisches Vorab’, was part of the Cluster of Excellence ‘Functional Biodiversity Research’.

## Contributions by the Authors

J.I. conceived the study design. U.P. performed the experiment, analysed the data and wrote the manuscript, which was then edited by J.I.

## Conflict of Interest Statement

None declared.
